# Radiosurgery-Based AVM Scale Is Proposed for Combined Embolization and Gamma Knife Surgery for Brain Arteriovenous Malformations

**DOI:** 10.3389/fneur.2021.647167

**Published:** 2021-03-30

**Authors:** Xiangyu Meng, Hongwei He, Peng Liu, Dezhi Gao, Yu Chen, Shibin Sun, Ali Liu, Youxiang Li, Hengwei Jin

**Affiliations:** ^1^Beijing Neurosurgical Institute, Capital Medical University, Beijing, China; ^2^Department of Interventional Neuroradiology, Beijing Tiantan Hospital, Capital Medical University, Beijing, China; ^3^Department of Gamma-Knife Center, Beijing Tiantan Hospital, Capital Medical University, Beijing, China; ^4^Department of Neurosurgery, Beijing Tiantan Hospital, Capital Medical University, Beijing, China; ^5^Beijing Engineering Research Center, Beijing, China

**Keywords:** BAVM, RBAS, endovascular embolization, gamma knife surgery, modified radiosurgery-based AVM score

## Abstract

**Background and purpose:** To evaluate whether a radiosurgery-based arteriovenous malformation (AVM) scale (RBAS) could be used to predict obliteration of brain arteriovenous malformations (bAVMs) supposed for combined endovascular embolization (EMB) and gamma knife surgery (GKS) treatment.

**Methods:** bAVM patients who underwent GKS with or without previous EMB from January 2011 to December 2016 at our institution were retrospectively reviewed. The patients were categorized into a combined treatment group and a GKS group. A 1:1 propensity score matching (PSM) was used to match the two groups. Pre-EMB and pre-GKS RBAS were assessed for every patient. Multivariate analysis was performed to find factors associated with complete obliteration in the combined treatment group. Survival analysis based on sub-groups according to RBAS was performed to compare obliteration rate and find cutoffs for appropriate treatment modalities.

**Results:** A total of 96 patients were involved, and each group comprised 48 patients. There was no difference between the two groups in terms of obliteration rate (75.0 vs. 83.3%, *p* = 0.174). Pre-EMB RBAS (*p* = 0.010) and the number of feeding arteries (*p* = 0.014) were independent factors associated with obliteration rate in the combined treatment group. For the combined treatment patients, sub-group analysis according to pre-EMB RBAS (score <1.0, 1.0–1.5, and >1.5) showed statistical difference in obliteration rate (*p* = 0.002). Sub-group analysis according to RBAS between the two groups showed that the obliteration rate of the GKS group is significantly higher than the combined group when RBAS >1.5 (47.4 vs. 66.7%, *p* = 0.036).

**Conclusions:** The RBAS is proposed to be efficient in predicting obliteration of bAVMs supposed to receive combined EMB and GKS treatment. Patients with RBAS >1.5 are inclined to be more suitable for GKS instead of the combined treatment.

## Introduction

Treatment of prior embolization (EMB) followed by gamma knife surgery (GKS) for brain arteriovenous malformation (bAVM) is still controversial. A combined approach is usually required for complicated bAVMs considering the limitation of each treatment modality. A combined approach with partial EMB will reduce the size or blood flow of nidus, delivering a proper dose for the remaining nidus (1). However, arguments still exist about whether EMB will reduce the obliteration rate after GKS as well as whether to select the combined treatment strategy for bAVM patients when monotherapy is available in the meantime. Advocates of EMB before GKS have agreed with the opinion that EMB can reduce the size of bAVMs and reduce the risk of hemorrhage by obliterating arteriovenous fistulas (AVFs) and intranidal aneurysms within bAVMs (2–5). But many researchers state that the combined treatment is not as effective as the single GKS treatment (6–8). The most common reason for EMB before GKS not being approved was that prior EMB will complicate the residual nidus and the embolic agents may block radiation from the GKS (9). Previous studies have compared the obliteration rate focusing on the pre-GKS angioarchitectural characteristics of the combined-treatment patients and GKS patients, while the pre-EMB characteristics were ignored (5, 6, 10, 11). Chen compared the outcomes of EMB and GKS vs. GKS alone for AVMs using pre-EMB malformation features. He concluded that EMB embolization negatively affecting obliteration of GKS is not tenable (12). The debate is still intense, and a combined treatment effect difference among different patient doses exists. To find suitable factors predicting obliteration for the combined treatment, as well as classify bAVMs suitable for combined treatment or GKS, the current study was performed using pre-EMB features.

## Methods

### Patients

A database including 319 cases of patients with bAVMs who underwent Leksell Gamma Knife (AB Elekta, Stockholm, Sweden) treatment between January 2011 and October 2016 at our institution was retrospectively reviewed. The combined treatment group comprised 48 consecutive patients with previous EMB. The GKS group was defined as bAVMs treated by GKS alone. A 1:1 propensity score matching (PSM) was used to match the GKS group with the same number and baseline data such as age, gender, hemorrhage, location of nidus, margin dose, maximum dose, volume, and radiosurgery-based AVM scale (RBAS) score as the combined group before EMB. All involved patients had more than 2 years clinical and radiological follow-up. Patients without complete clinical information who underwent fractionated stereotactic radiosurgery or refused to participate were excluded. Patients' baseline information, pre-treatment angioarchitectural characteristics, EMB and GKS details, complications, and clinical outcomes were collected. This study obtained ethical approval from the institutional review board, and all patients signed an informed consent.

### Definition of Parameters

**Figure 1:**
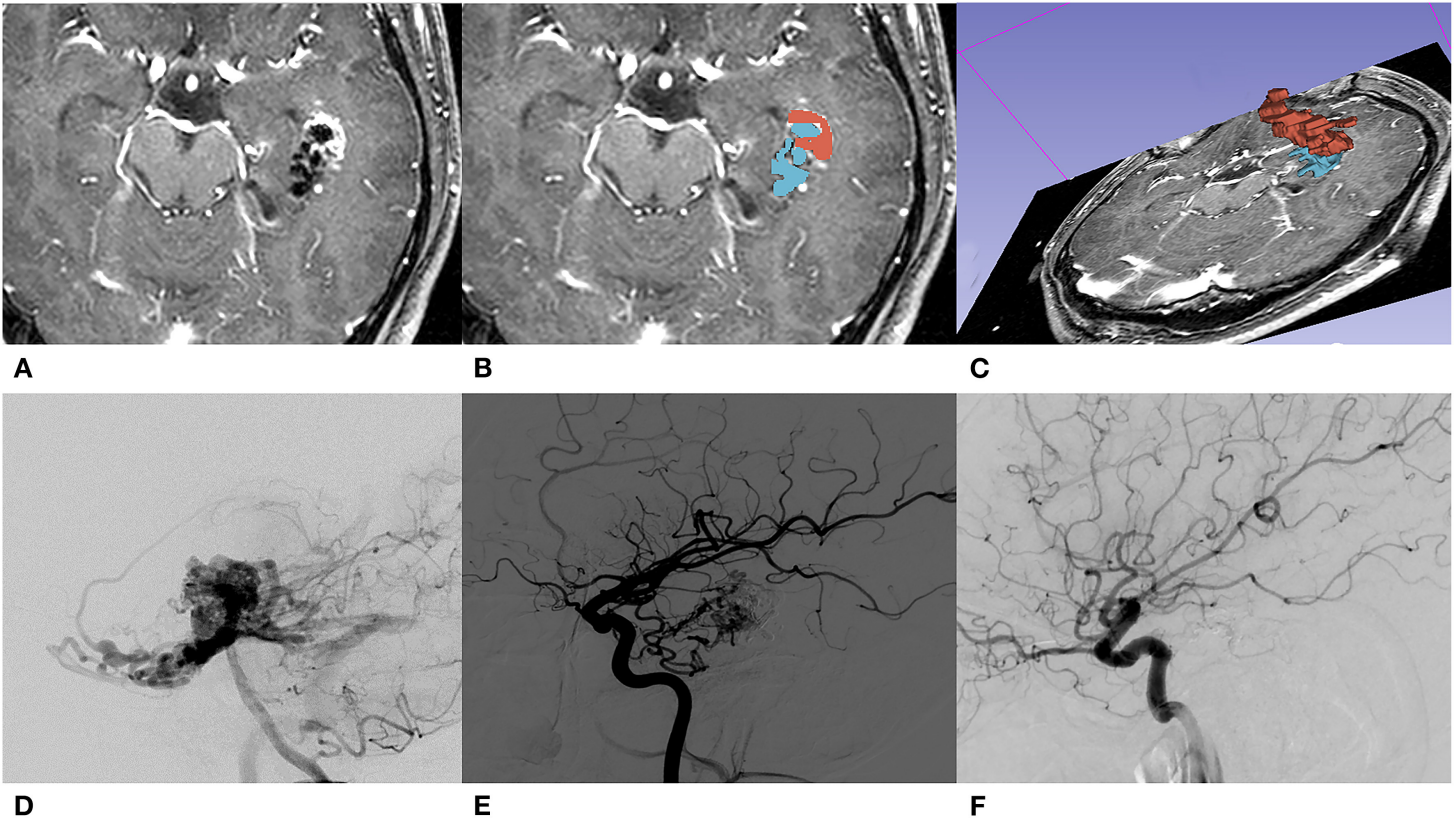
A 31-year-old male with initial presentation of epilepsy. **(A)** The embolized AVM located in the left temporal lobe is shown on T1 contrast-enhancement series of pre-GKS 3D-time-of-flight (TOF) MRI. **(B)** Segmentations of nidus (red) and embolic agent (blue) were both manually contoured slice by slice. **(C)** A 3D reconstruction of manually contoured region of interest (ROI) in a 3D slicer. **(D)** Pre-EMB angiography of the left vertebral artery; the nidus was mainly fed by the left posterior cerebral artery. **(E)** After embolization, branches of the left middle carotid artery feed the residual nidus. **(F)** DSA performed 36 months after GKS showed that the nidus was totally obliterated.

The location of lesions was classified into three categories according to RBAS (13). The sensorimotor, language and visual cortex, thalamus, internal capsule, brainstem, cerebellar peduncles, and deep cerebellar nuclei are considered eloquent areas. The number of feeding arteries and draining veins were categorized into single and multiple. The venous pattern is considered deep if any of the drainage is through deep veins (such as the internal cerebral veins, basal veins, or precentral cerebellar veins). The diameter of the feeding artery is assessed at a distal segment of the arterial pedicle within 1 cm of the nidus (14). The volume of the nidus was measured manually using a 3D slicer (an open-source software, version 4.10.2) platform on T1 contrast-enhancement sequence slice by slice. In order to maintain the consistency of the data obtained, the pre-EMB nidus volumes were also obtained on pre-GKS MRI and calculated by adding residual nidus volume and embolic agent's volume (Figure 1). All the volumes were segmented by two experienced interventional neuroradiologists using a 3D slicer.

### Endovascular EMB and GKS

The aim of pre-GKS EMB was the reduction of bAVM volume and prevention of bleeding or rebleeding. Decision making was based on consensus by multidisciplinary meeting comprising at least three experienced senior interventional neuroradiologists. Endovascular treatment was performed under general anesthesia, and a biplane angiography system was used (Siemens, Germany or Philip, Netherland). All procedures were performed according to standardized procedures of our institution for interventional EMB. The liquid embolic materials included N-butyl cyanoacrylate (NBCA) and copolymer ethylene vinyl alcohol (Onyx; Medtronic, Irvine, California, CA, USA).

Stereotactic frame placement and stereotactic planning neuroimaging was performed for each patient. The acquired images were transferred to the Leksell Gamma-Plan workstation (Elekta AB, Elekta Company, Stockholm, Sweden) for the definition and dose planning. The treatment target was delineated by T1 contrast-enhancement sequence and T2 sequence on 3D stereotactic MRI. Dose planning was performed by neurosurgeons and medical physicists depending on bAVM nidus location and volume. The resolution of T1 contrast-enhancement sequence was 512 × 512 mm, and the thickness of each slice was 2.00 mm. All voxels were 0.47 × 0.47 × 2.00 mm.

### Complications and Clinical Outcome

EMB-related complications were divided into hemorrhagic and ischemic types. Hemorrhagic complication is defined as sudden onset of symptoms (such as headache, vomiting, and loss of consciousness) and intracranial hemorrhage confirmed by CT. Ischemic complication is defined as new neurofunctional deficits and infarctions confirmed by CT or MRI. GKS-related complications included postoperative hemorrhage and symptomatic radiation-induced change (RIC, includes cystic change, edema, or atypical T2 increasing around the treated nidus). Scheduled clinical and imaging follow-up were performed for each patient. All patients received MRI examination every 6 months during the first year after GKS treatment, which were then turned into yearly until the nidus disappeared, followed by digital subtraction angiography (DSA) confirmation. Two senior neuroradiologists evaluate the last radiographic follow-up independently, and a third one will reevaluate if the result is controversial. Complete obliteration of bAVM was defined as no contrast filling of preexistent nidus and the absence of early venous drainage on DSA, or as an absence of flow voids on T1- and T2-weighted images on MRI (15). Favorable clinical outcome is defined as mRS ≦ 2, and unfavorable clinical outcome is defined as mRS ≧ 3. An excellent outcome consisted of complete nidus obliteration and no development of new neurological deficits.

### Statistical Analysis

Descriptive statistics are presented as median/mean for continuous variables and as frequency and percentage for categorical variables. Continuous variables were compared using a *t*-test, and categorical variables were analyzed by a chi-square test. Kaplan–Meier and COX regression analysis was performed, respectively, for univariate and multivariate variables predicting complete obliteration. The inclusion standard for multivariate analysis is *p* < 0.1 in univariate analysis. The significance of all statistical tests was defined as *p* < 0.05. The PSM was performed with R software (version 3.6.1). All statistical analysis was conducted using SPSS (version 19.0, IBM) and Graphpad software (Version 7.0, Graphpad Inc.).

## Results

### Demographics and Clinical Characteristics

The cohort consists of 48 patients who underwent combined treatment and 48 patients who underwent GKS. There are 39 males and 57 females, and age ranges from 7 to 59 years old (mean 26.53 ± 1.29 years old). Initial presentations include 64 (66.7%) hemorrhage and 19 (19.8%) seizures. Thirty-two (33.3%) lesions were located at the frontal or temporal lobe, 46 (47.9%) were located at the parietal, occipital, intraventricular, corpus callosum, and cerebellar areas, and 18 (18.8%) were located at the basal ganglia, thalamus, or brainstem. The distribution of Spetzler–Martin (SM) scale was grade I in 12 (12.5%) patients, II in 34 (35.4%) patients, III in 34 (35.4%) patients, IV in 13 (13.5%) patients, and V in 3 (3.2%) patients. The mean volume of the combined treatment patients was 7.50 ± 1.32 and 4.80 ± 0.71 cm^3^ for pre-EMB and post-EMB, respectively. There is no statistical difference in terms of baseline and clinical information between the combined treatment group and the GKS group. A total of 59 EMB procedures were performed in 48 patients. Nine patients underwent two procedures, and one of them underwent four procedures. Onyx was used in 46 patients (95.8%), NBCA in 2 (4.2%) patients, and both in 2 (4.2%) patients. The mean reduction of the nidus volume was 2.70 cm^3^, and mean reduction of the RBAS and modified RBAS (mRBAS) were both 0.27. A total of 96 GKS procedures were performed. The mean prescription dose was 16.75 ± 0.14 Gy (range 14–20 Gy). The mean maximum dose was 33.16 ± 0.25 Gy (range 28–38 Gy). Demographics and clinical characteristics are listed in Table 1.

**Table 1:** Matched patients' clinical and radiographical parameters.

**Parameters**	**Total**	**EMB+GKS**	**GKS**	***p***
	***n* = 96**	***n* = 48**	***n* = 48**	
Age (mean ± SD, years)	26.5 ± 1.3	27.5 ± 1.9	25.6 ± 1.8	0.466
Gender (*n*, %)				0.146
Male	39 (40.6)	23 (47.9)	16 (33.3)	
Female	57 (59.4)	25 (52.1)	32 (66.7)	
Rupture (*n*, %)	64 (66.7)	29 (60.4)	35 (72.9)	0.279
Nidus location (*n*, %)				0.385
Frontal or temporal lobe	32 (33.3)	13 (27.1)	19 (39.6)	
Brainstem, basal ganglia, or thalamic	18 (18.8)	9 (18.7)	9 (18.7)	
Parietal, occipital, intraventricular, corpus callosum, or cerebellar	46 (47.9)	26 (54.2)	20 (41.7)	
Locations of draining veins (*n*, %)				0.538
Superficial	43 (44.8)	20 (41.7)	23 (47.9)	
Deep	53 (55.2)	28 (58.3)	25 (52.1)	
Volume (pre-EMB, mean ± SD, cm3)	6.30 ± 0.82	7.50 ± 1.33	5.10 ± 0.94	0.144
Volume (pre-GKS, mean ± SD, cm3)	4.95 ± 0.58	4.80 ± 0.71	5.10 ± 0.94	0.798
mRBAS scores (pre-EMB, mean ± SD)	1.26 ± 0.09	1.39 ± 0.14	1.12 ± 0.10	0.110
mRBAS scores (pre-GKS, mean ± SD)	1.12 ± 0.07	1.12 ± 0.08	1.12 ± 0.10	0.954
RBAS scores (pre-EMB, mean ± SD)	1.42 ± 0.09	1.58 ± 0.14	1.26 ± 0.11	0.071
RBAS score (pre-GKS, mean ± SD)	1.28 ± 0.07	1.30 ± 0.08	1.26 ± 0.11	0.747
Prescription dose (mean ± SD, Gy)	16.75 ± 0.14	16.77 ± 0.20	16.73 ± 0.20	0.882
Maximum dose (mean ± SD, Gy)	33.16 ± 0.25	33.35 ± 0.36	32.98 ± 0.34	0.453
Obliteration (*n*, %)	76 (79.2)	36 (75.5)	40 (83.3)	0.452
Complications (*n*, %)				
Hemorrhage	2 (2.1)	1 (2.1)	1 (2.1)	1.00
Permanent neurofunctional deficits	2 (2.1)	1 (2.1)	1 (2.1)	1.00
Favorable clinical outcome (*n*, %)	96 (100.0)	48 (100.0)	48 (100.0)	1.00
Excellent clinical outcome (*n*, %)	76 (79.2)	36 (75.5)	40 (83.3)	0.452

*GKS, gamma knife surgery; EMB, endovascular embolization; SD, standard deviation; RBAS, radiosurgery-based arteriovenous malformations grading system; mRBAS, modified radiosurgery-based arteriovenous malformations grading system*.

### Factors Affecting the Obliteration Rate of Combined Treatment Patients

The mean radiologic follow-up time was 45.13 months. Total obliteration was achieved in 76 (76/96, 79.2%) patients. There is no statistical difference in obliteration rate between the two groups (75.0 vs. 83.3%, *p* = 0.452). In the combined treatment group, the history of hemorrhage, number of feeding arteries, pre-EMB nidus volume, pre-EMB RBAS score, and pre-EMB mRBAS score showed significant difference in univariate COX analysis for obliteration rate. Multivariate COX regression analysis showed that pre-EMB RBAS score (*p* = 0.010) and number of feeding arteries (*p* = 0.014) were independent factors associated with obliteration rate (Table 2). Twenty-one patients have DSA confirmation in the combined treatment group, and 16 achieved obliteration. Of the five patients without obliteration, all had an RBAS over 1.5.

**Table 2:** COX regression analysis of factors associated with obliteration in combined treatment group.

**Parameters**	**Univariate analysis**			**Multivariate analysis**		
	**HR**	**95%CI**	***p***	**HR**	**95%CI**	***p***
Age	0.983	0.955–1.012	0.242	–	–	–
Gender	1.116	0.572–2.179	0.748	–	–	–
Rupture	0.407	0.188–0.881	0.023^*^	0.643	0.265–1.561	0.329
Nidus location	0.881	0.380–2.042	0.768	–	–	–
Single/multiple feeding	0.277	0.091–0.845	0.024^*^	4.073	1.330–12.473	0.014^*^
Diameter of feeding artery	0.884	0.664–1.175	0.395	–	–	–
No. of draining veins	1.595	0.794–3.204	0.190	–	–	–
Speztler–Martin grades	0.736	0.503–1.077	0.115	–	–	–
Deep/superficial draining veins	0.965	0.490–1.901	0.918	–	–	–
Companies with AVF	0.555	0.212–1.450	0.229	–	–	–
Pre-EMB volume	0.932	0.872–0.996	0.038^*^	1.094	0.944–1.267	0.233
Pre-EMB RBAS scores	0.451	0.241–0.841	0.012^*^	0.433	0.230–0.816	0.010^*^
Pre-EMB mRBAS scores	0.488	0.276–0.865	0.014^*^	1.985	0.106–37.233	0.647
Prescription dose	1.124	0.862–1.464	0.388	–	–	–
Maximum dose	1.113	0.949–1.305	0.187	–	–	–
Isodose curve	0.967	0.777–1.203	0.760	–	–	–

*GKS, gamma knife surgery; EMB, embolization treatment; HR, hazard ratio; CI, confidence interval; RBAS, radiosurgery-based arteriovenous malformations grading system; mRBAS, modified radiosurgery-based arteriovenous malformations grading system*.

* *Significant difference in statistical analysis*.

### Sub-Group Analysis According to RBAS Scale

**Figure 2:**
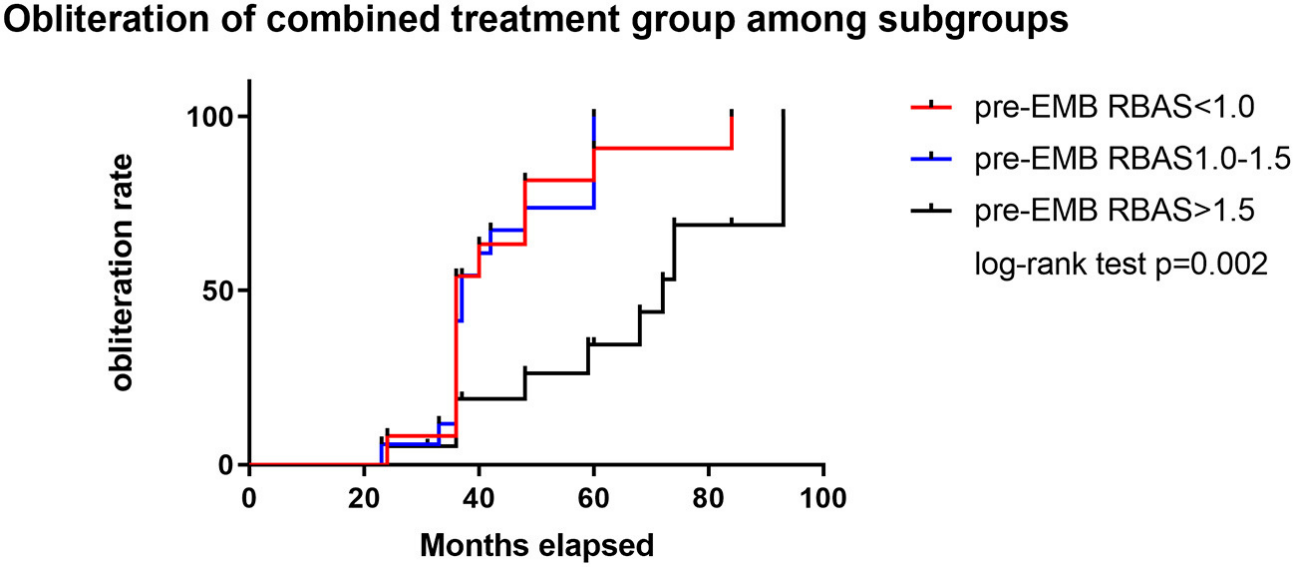
Subgroup analysis of RBAS before EMB for the combined treatment group. Kaplan–Meier analysis showed significant difference between subgroups in obliteration rate; RBAS > 1.5 is significant with lower obliteration rate than the other two groups (log-rank test, *p* = 0.002).

**Figure 3:**
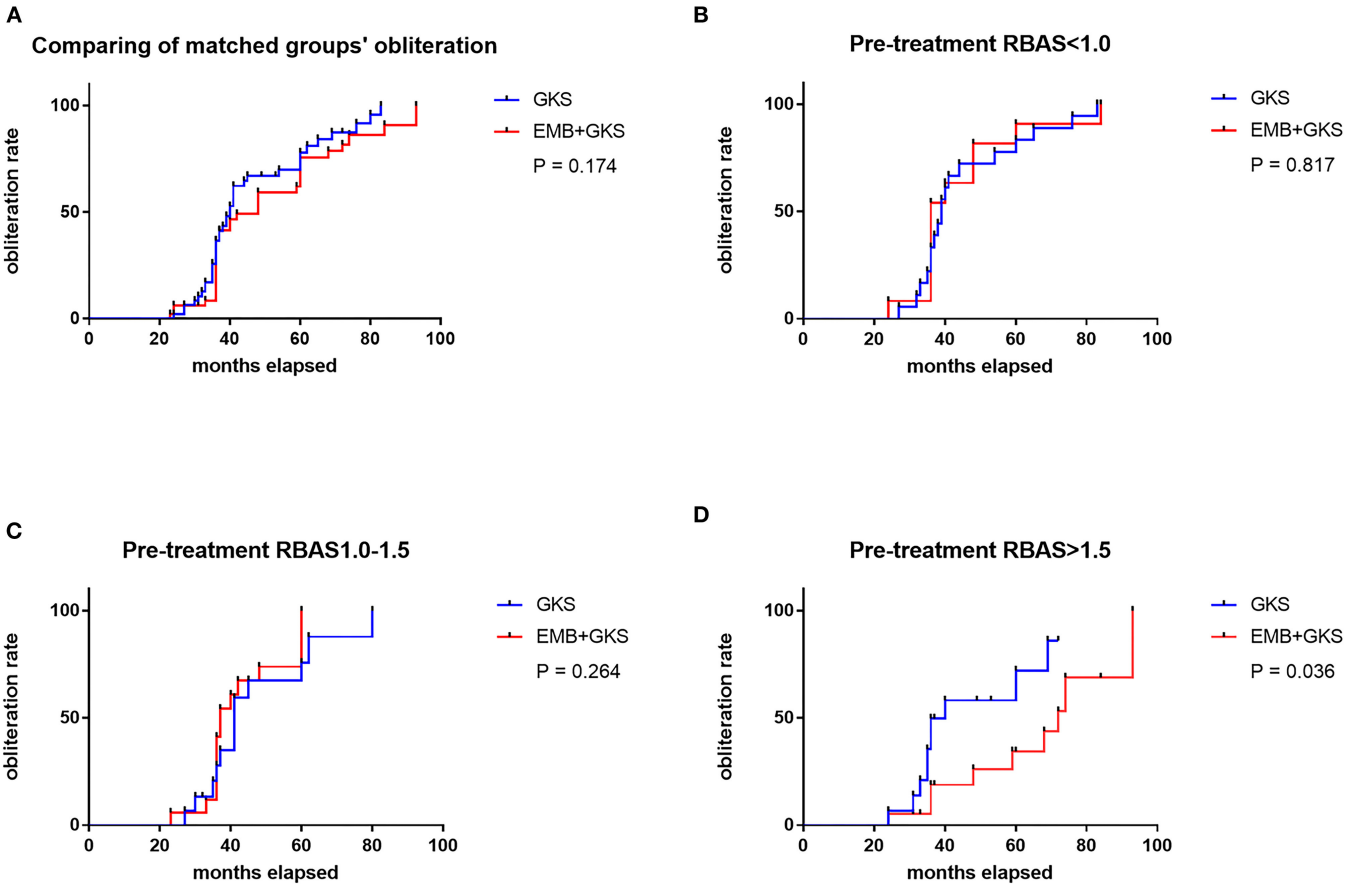
**(A)** Comparison of obliteration rate between groups by Kaplan–Meier analysis. No significant difference was found between the two treatment modalities (75.0 vs. 83.3%, *p* = 0.174). **(B–D)** Comparison of obliteration rate between sub-groups; the obliteration rate of GKS group is statistically higher than the combined treatment group when pre-treatment RBAS > 1.5 (47.4 vs. 66.7%, *p* = 0.036).

A cutoff of 1 and 1.5 points in pre-GKS RBAS was identified as the most informative cutoff from our series. Before EMB, Kaplan–Meier analysis showed the obliteration rate was significantly different between the RBAS subgroups in the combined treatment group, and RBAS > 1.5 is related with lower obliteration rate (log-rank test, *p* = 0.002, Figure 2). In Kaplan–Meier analysis, there is no statistical difference in obliteration rate between the two groups (*p* = 0.172, Figure 3A). The obliteration rate between subgroups according to RBAS (RBAS <1.0, 1.0–1.5, and >1.5) was also compared (Figures 3B–D). Of the different RBAS scores, the obliteration rate was statistically different between the combined treatment group and the GKS group. Patients with RBAS > 1.5 have lower obliteration in the combined treatment group (47.4 vs. 66.7%, *p* = 0.036, Figure 3D). We also performed statistical analysis for the GKS-only group. RBAS is originally proposed for evaluating bAVM patients undergoing GKS in terms of clinical outcomes. The result of our analysis showed that there is significant statistical difference between obliteration and non-obliteration patients (*p* < 0.01) in the GKS group, indicating that as the RBAS increases, the obliteration decreases.

### Complications and Follow-Up

The mean clinical follow up time was 60.4 months (range from 24 to 98 months). Of the patients who received combined treatment, 59 EMB procedures were implemented, and 6 (10.2%) procedure-related adverse events happened, including 1 hemorrhagic and 5 ischemic types. One patient suffered subarachnoid hemorrhage 1 day after EMB, in which we considered that hemodynamic changes caused normal perfusion pressure breakthrough. The patient eventually recovered fully (mRS = 0). One patient suffered permanent contralateral limb weakness (mRS = 1 at last follow-up). Four patients experienced transient ischemic symptoms (one limb weakness, two limb numbness, and one blurred vision) and recovered fully at discharge. All patients had favorable clinical outcome (mRS < 2), and 36 in 48 (75.0%) patients had excellent clinical outcome in the combined treatment group.

In the GKS group, a total of 3 GKS-related adverse events occurred. One patient suffered hemorrhage (presented as minor headache, micro-bleeding confirmed by MRI). The patient presented as weakness in left limb; mRS score was 1 at last follow-up. The other two complications include one with increased epilepsy with edema and one limb weakness with atypical increased T2 signal. All patients had favorable clinical outcome (mRS < 2), and 40 in 48 (83.3%) patients had excellent clinical outcome in the GKS group. There is no statistical difference in complication rate and clinical outcomes between the two groups (Table 1).

## Discussion

Many researchers proposed that EMB prior to GKS may reduce the final obliteration rate (7, 9, 11, 16). Several potential mechanisms have been suggested for the diminished obliteration rate of GKS induced by prior EMB, including superimposition of embolic material (17), the presence of collateral feeding vessels (18), and shielding effect caused by EMB materials with high atomic mass. Someone held that EMB may induce hypoxia, making bAVM tissue less radiosensitive and increasing its angiogenic activity (19). However, Mamalui-Hunter et al. (20) and Schlesinger et al. (21) confirmed that both Onyx and NBCA had negligible dose perturbation of GKS treatment by constructing physics modeling. Chen et al. (12) refuted the prevalent notion that AVM EMB negatively affects the likelihood of obliteration after GKS. There is no statistical difference in terms of final obliteration rate between the combined treatment patients and the GKS patients in this cohort. A combination of EMB and GKS for bAVMs will not decrease the obliteration rate compared with GKS alone. In fact, although the pre-EMB and pre-GKS volumes in this study are not significantly different, there is a tendency for a larger volume in the combined treatment group (7.50 vs. 5.10 cm^3^), suggesting that the combined treatment group has somewhat more complex/larger AVMs. Thus, similar outcomes suggest a beneficial effect for EMB, though not statistically significant.

GKS was commonly used as a supplementary treatment to EMB. The Pittsburgh RBAS were developed by multivariate analysis to predict bAVM obliteration without new neurological deficit after GKS (13, 22, 23). Compared with the Virginia score (24) and SM scale, RBAS score was more applicable to the prognosis of GKS and has been widely accepted. Subsequently, the mRBAS score was also proposed by Pollock and Flickinger, proving to be more convenient and accurate in predicting the prognosis of patients after GKS treatment (25). This modified score changed the location groups of the nidus into two categories. Deep location was defined as basal ganglia, thalamus, or brainstem, and others were all classified into the contrary. However, there are still no corresponding scores and predictors that could be used for preoperative evaluation for EMB combined with GKS. In the current study, we calculated the RBAS scores, mRBAS scores, and other angioarchitecture characteristics before EMB and found two factors that can preliminarily affect the prognosis of the combined treatment. RBAS is an independent factor affecting the final obliteration rate for the combined treatment. When RBAS > 1.5, the obliteration of bAVMs decreased. Another independent factor affecting the obliteration rate is the number of feeding arteries. A nidus with a single feeder is easier to be completely occluded. The number of feeding arteries was one factor preserved in the Puerto Rico scale, Buffalo scale, and AVMES scale, which are proposed for predicting clinical outcomes of bAVMs undergoing endovascular treatment (14, 26, 27). To further explore which patient is more suitable for combined treatment or GKS, sub-group analysis according to RBAS was performed. The result showed that the occlusion rate of patients with RBAS > 1.5 decreased in the combined treatment group, indicating that patients with higher preoperative RBAS score are not suitable for combination therapy. On the contrary, GKS alone will be more beneficial to these patients. For patients with RBAS ≤ 1.5, the combined treatment and GKS will perform equally. The use of the RBAS scale should be considered by physicians who counsel bAVM patients about outcomes following GKS as well as combined management. The combination of RBAS score and number of arteries is suitable and an explicable factor for the prognosis of combined treatment for bAVMs.

It is known that aggressive EMB of a large bAVM in a single session can be associated with high rate of periprocedural hemorrhage. The indications for combined management in our own series including reduction of the nidus volume to facilitate further GKS and possible prevention of bleeding or rebleeding by EMB of fistulas or intranidal aneurysms. In this series, no major neurologic deterioration was marked after EMB, reflecting careful case selection and non-aggressive strategy of endovascular management. The benefits of EMB before GKS include reducing bAVM volume, allowing application of higher radiation dose to the margin of the smaller target volume with a better obliteration rate and fewer complications (28). In our series, we reduced about 36.0% of the volume, making the target volume of GKS smaller, and the dosage applied on residual nidus may increase. Secondly, EMB can occlude associated arterial or intranidal aneurysms to reduce the risk of bleeding when awaiting the delayed action of GKS and bAVM thrombosis. Pre-EMB can also target large AVFs associated with plexiform-shaped bAVMs, which are less sensitive to GKS (29), and reduce symptoms associated with arterial steal or venous hypertension. In this cohort, the total hemorrhage rate is 3.1% with an obliteration of 79.2%. There is no significance in terms of total hemorrhage rate (2.1 vs. 2.1%, *p* = 1.000), permanent neurofunctional deficits (2.1 vs. 2.1%, *p* = 1.000), and excellent clinical outcomes (75.0 vs. 83.3%, *p* = 0.452) between the two groups. The use of combined treatment could be carefully considered for patients with RBAS score <1.5. A lower obliteration rate observed in embolized bAVMs followed by GKS was related with the patient selection.

One limitation is inevitable in the current study inherent to its single-center and retrospective property, including population and selection bias, especially the strategy of pre-GKS EMB, which may vary among centers since there are no guidelines. Another limitation is that not all patients had DSA to confirm obliteration. We ensured that two senior neuroradiologists evaluate the last radiographic follow-up independently and a third one will reevaluate if the result is controversial to assure the accuracy of the result. Twenty-one patients have DSA confirmation in the combined treatment group, and 16 achieved obliteration. Of the five patients without obliteration, all had an RBAS > 1.5, indicating that there is a tendency that RBAS could be used to predict obliteration of the combined treatment patients even though we used DSA for confirmation. Further studies involving a multi-center and larger number of cases with DSA confirmation are necessary to confirm the conclusions.

## Conclusions

RBAS is proposed to be efficient in predicting obliteration of bAVMs supposed to receive combined EMB and GKS treatment. Patients with RBAS > 1.5 are inclined to be more suitable for GKS instead of combined treatment.

## Data Availability Statement

The raw data supporting the conclusions of this article will be made available by the authors, without undue reservation.

## Ethics Statement

The studies involving human participants were reviewed and approved by Medical Ethics Committee of Beijing Tiantan Hospital. Written informed consent to participate in this study was provided by the participants' legal guardian/next of kin.

## Author Contributions

This study was designed by YL, AL, HH, and HJ. Material preparation and data collection and analysis were performed by DG, HJ, SS, PL, XM, and YC. The first draft of the manuscript was written by XM. All authors commented on previous versions of the manuscript. All authors read and approved the final manuscript.

## Conflict of Interest

The authors declare that the research was conducted in the absence of any commercial or financial relationships that could be construed as a potential conflict of interest.
